# Supplementary material for: Understanding LRRK2 kinase activity in preclinical models and human subjects through quantitative analysis of LRRK2 and pT73 Rab10
Source: Sci Rep. 2021 Jun 18;11:12900. doi: 10.1038/s41598-021-91943-4 (PMC8213766; doi:10.1038/s41598-021-91943-4)
Supplement: Supplementary file 1 — Supplementary Information. [file 41598_2021_91943_MOESM1_ESM.pdf]

Supplementary Information for: Understanding LRRK2 kinase activity in preclinical models and human subjects through quantitative analysis of LRRK2 and pRab10

Xiang Wang, Elvira Negrou, Michael T. Maloney, Vitaliy V Bondar, Shan V. Andrews, Manuel Montalban, Ceyda Llapashtica, Romeo Maciuca, Hoang Nguyen, Hilda Solanoy, Annie Arguello, Laralynne Przybyla, Nathan J. Moerke, Sarah Huntwork-Rodriguez, Anastasia G. Henry*

*To whom correspondence should be addressed: henry@dnli.com

Affiliations:

Denali Therapeutics, Inc., 161 Oyster Point Blvd., South San Francisco, CA 94080

Supplementary Table 1

Assay	LLOD (ng/mL)	LLOQ (ng/mL)	CV (%)	Linear range (ng/mL)
pS935 LRRK2	0.06	0.14	14.7	0.16-600
LRRK2	0.03	0.07	15.8	0.06-96

Supplementary Table 1: Attributes of the pS935 LRRK2 and LRRK2 MSD assays as assessed using recombinant LRRK2 protein, including LLOD, lower limits of quantification (LLOQ), coefficient of variation (CV) and linear range. The LLOD was calculated based on identifying the signal that was three times the standard deviation of the background plus average of the background, and the LLOQ was calculated based on ten times the standard deviation of the background plus average of the background.

Supplementary Table 2

Assay	LLOD (ng/mL)	LLOQ (ng/mL)	CV (%)	Linear range (ng/mL)
pRab10	8.36	58.56	16.3	65.2-8350
Rab10	0.02	0.07	11.8	0.56-45.7

Supplementary Table 2: Attributes of the pRab10 and Rab10 MSD assays as assessed using recombinant Rab10 protein, including LLOD, LLOQ, CV, and linear range.

Supplementary Table 3

The effects of LRRK2 variants on LRRK2 activity

	HEK293 OE cells (**a**)		primary mouse astrocytes (**b**)			human PBMC (carrier Vs non-carrier) (**c**)		
Genotype	pRab10		pRab10	pS935 LRRK2		pS935 LRRK2	LRRK2	pRab10/LRRK2
LRRK2 G2019S	2.7		1.78	0.64		0.65	0.69	1.44

	HEK293 OE cells (**d**)			KI A549 cells (**e**)		human PBMC (carrier Vs non-carrier) (**f**)		
Genotype	LRRK2	pRab10		LRRK2	pRab10		pRab10	pRab10/LRRK2
LRRK2 N551K R1398H	0.3	0.4		0.47	0.5		0.66	0.66

The effects of lysosomal and immune stressors in iPSC-derived microglia (**g**)

	chloroquine (50 μM)			Bafilomycin A1 (100 nM)			IFNγ (20 ng/mL)	
	6 hours	24 hours		6 hours	24 hours		6 hours	24 hours
LRRK2	no change	no change		1.4	1.2		no change	7
pRab10	3.5	3.1		9.1	8		2	12.9

Supplementary Table 3: Summary of the effects of LRRK2 genetic variants and different treatments on the LRRK2 level and activity.

Data described throughout our studies are summarized here and fold changes are shown (**a**) based on the mean values from Supplementary Fig. S7A (**b**) based on the mean values from Fig.

3B; (**c**) based on the relative geometric mean between carrier vs non-carrier in the PD population from Fig. 5; (**d**) based on the mean values from Fig. 6B-C; (**e**) based on the mean values from Fig. 6D-E; (**f**) based on the standard deviation decrease in carriers vs non-carriers from Fig. 7; (**g**) based on the mean values from Fig. 3D-G and from Supplementary Fig.S5

Supplementary Table 4

Assay	LLOD (µg/mL)	LLOQ (µg/mL)	CV (%)	Linear range (µg/mL)
pS935 LRRK2	6	13.5	9.0	12~*3070
LRRK2	3.2	6	7.5	12~*3070

*highest lysate concentration tested

Supplementary Table 4: Attributes of the LRRK2 and pS935 LRRK2 MSD assays in human PBMC lysates, including LLOD, LLOQ, CV, and linear range.

Supplementary Table 5

Assay	LLOD (µg/mL)	LLOQ (µg/mL)	CV (%)	Linear range (µg/mL)
pRab10	48.5	191.9	12.4	191.9~*3070
Rab10	8.5	24	9.8	3-12

*highest lysate concentration tested

Supplementary Table 5: Attributes of the pRab10 and Rab10 MSD assays in human PBMC lysates, including LLOD, LLOQ, CV, and linear range.

Supplementary Table 6

Assay	LLOD (dilution factor)	LLOQ (dilution factor)	CV (%)	Linear range (dilution factor)
pS935 LRRK2	293	145	13.9	2-512
LRRK2	512	295	9.2	2-512

Supplementary Table 6: Attributes of the LRRK2 MSD assay in human whole blood, including LLOD, LLOQ, and linear range (assessed via dilution factor of whole blood) and CV.

Supplementary Table 7

	Model 1<br>Phenotype ~ Sex + Age + PC1 + PC2			Model 2<br>Phenotype ~ Sex + Age + PC1 + PC2 + Disease			Model 3<br>Phenotype ~ Sex + Age + PC1 + PC2 + G2019S			Model 4<br>Phenotype ~ Sex + Age + PC1 + PC2 + Disease + G2019S		
Phenotype	Estimate	SE	P	Estimate	SE	P	Estimate	SE	P	Estimate	SE	P
total LRRK2	0.196	0.28	0.485	0.171	0.28	0.5435	0.194	0.28	0.4888	0.171	0.28	0.5435
pRab10	-0.657	0.275	0.0182	-0.594	0.276	0.0332	-0.649	0.275	0.0198	-0.569	0.276	0.0416
pS935 LRRK2	0.103	0.281	0.7129	0.052	0.281	0.852	0.104	0.281	0.712	0.058	0.281	0.8378
pRab10/LRRK2	-0.663	0.275	0.0172	-0.566	0.277	0.0424	-0.643	0.275	0.0209	-0.541	0.277	0.0529

Supplementary Table 7: Linear regression statistics for association testing of LRRK2 and pRab10 levels against N551K R1398H haplotype status.

Raw protein levels were natural log transformed, and then fit in a linear model against the covariates listed under each “Model”. Residuals from each model were then inverse normal transformed and tested for association against the N551K R1398H haplotype variable. Estimate = standard deviation change in adjusted and transformed protein level for haplotype carriers relative to non-carriers. SE = standard error of estimate.

Supplementary Figure S1

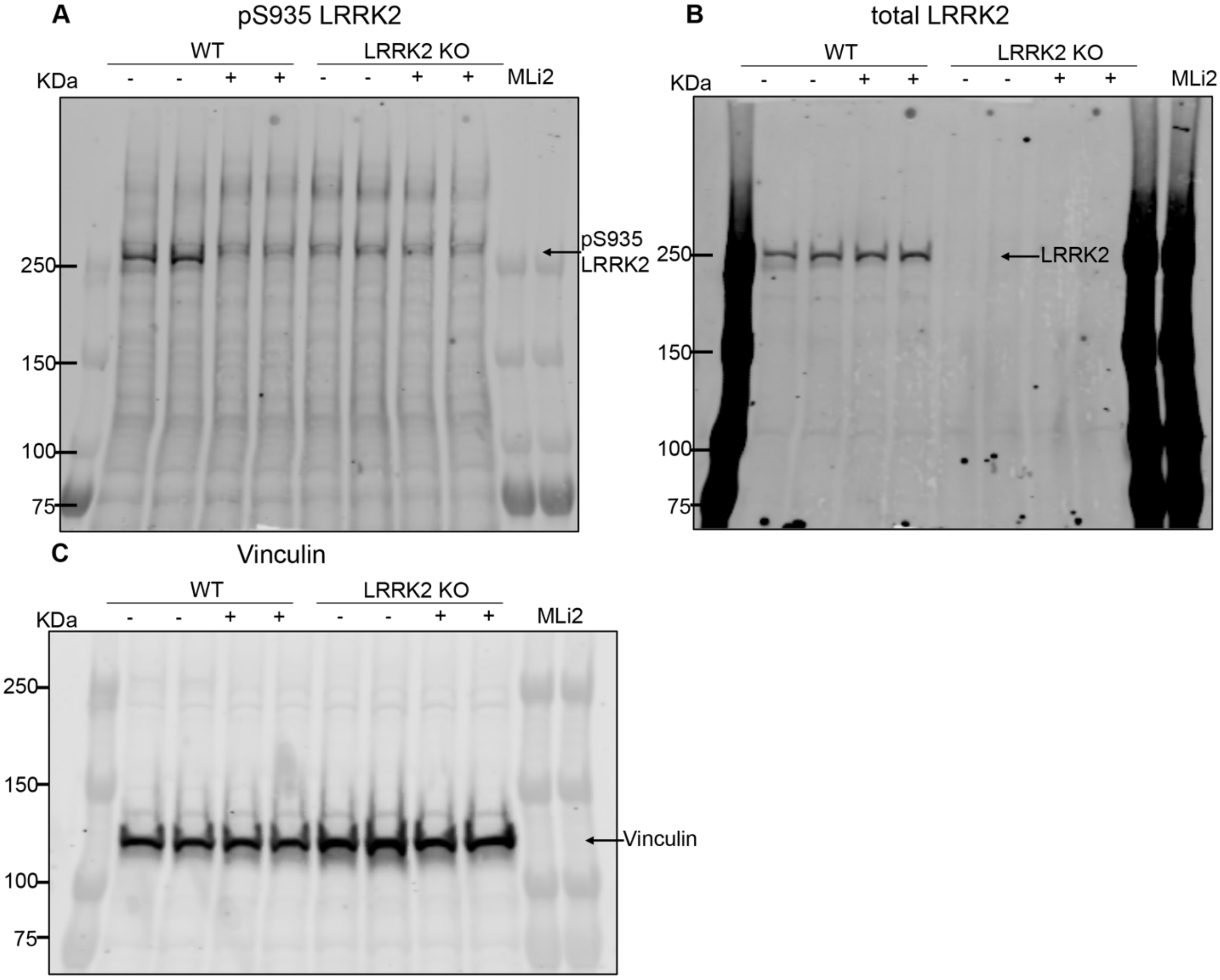

Supplementary Figure S1. pS935 LRRK2 and total LRRK2 levels measured in WT and *LRRK2* KO A549 cells assessed with western blot analysis.

Samples were run on NuPAGE 3-8% Tris-Acetate gels, and the membranes were cut around 75 kDa based on protein standards (Bio-Rad #1610374). The membranes were first incubated with rabbit anti-pS935-LRRK2 antibody (with secondary 800CW anti-rabbit antibody) and mouse anti-LRRK2 antibody (with secondary 680RD anti-mouse antibody). Images are shown at a high exposure (**A and B**). The membrane was then incubated with rabbit anti-vinculin antibody (with secondary 680RD anti-rabbit antibody) for loading control, and the image is shown with low exposure (**C**).

Supplementary Figure S2

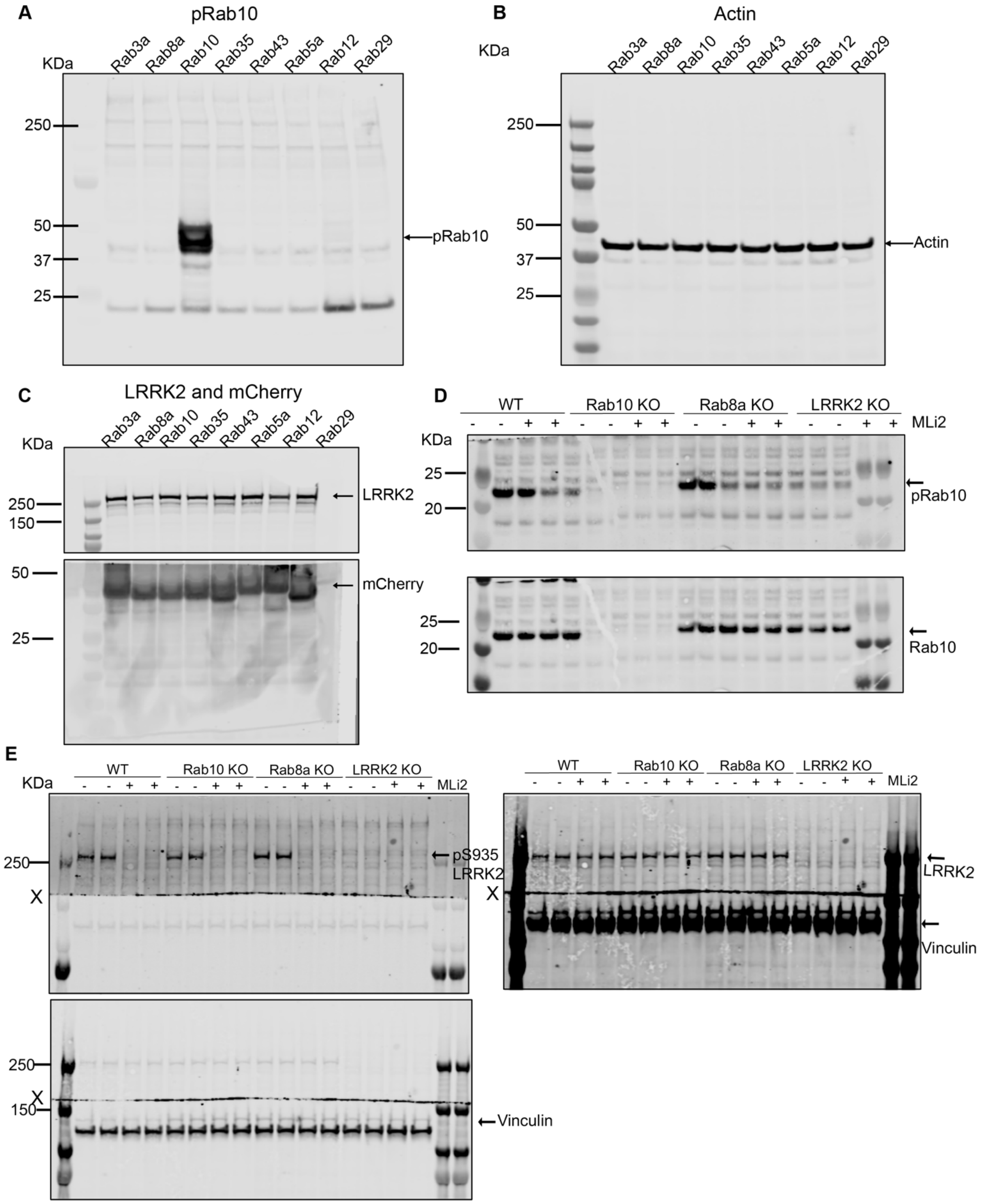

Supplementary Figure S2. The phospho-specific pT73 Rab10 antibody selectively and specifically detects Rab10 phosphorylation in cells as confirmed by western blot analysis.

A-C): The same sample set was run on duplicate gels (NuPage 4-12% Bis-Tris gels). For one set, the protein transferred membrane was incubated with rabbit anti-pRab10 antibody (**A**) and mouse anti-actin antibody (**B**). The expected size of mCherry-Rab10 is ~50 kDa. For the other set, the protein transferred membrane was cut around 75 kDa. The top membrane was incubated with mouse anti-LRRK2 antibody, and the bottom membrane was incubated with rabbit anti-mCherry antibody (**C**). **D-E)** The same samples were run on a NuPAGE 4-12% Bis-Tris gel to better resolve the Rab10 band (**D**) or a NuPAGE 3-8% Tris-Acetate gel to better resolve the LRRK2 band (**E**), respectively. For the Rab10 gel (**D**), the protein transferred membrane was cut around 37 kDa, and the bottom membrane was incubated with rabbit anti-pRab10 antibody and mouse anti-Rab10 antibody. For the LRRK2 gel (**E**), the protein transferred membrane was cut around 150 kDa (marked with "X"). The top membrane was incubated with rabbit anti-pS935-LRRK2 antibody (with secondary 800CW anti-rabbit antibody) and mouse anti-LRRK2 antibody (with secondary 680RD anti-mouse antibody), and the bottom membrane was incubated with rabbit anti-vinculin antibody (with secondary 680RD anti-rabbit antibody) as a loading control.

Supplementary Figure S3

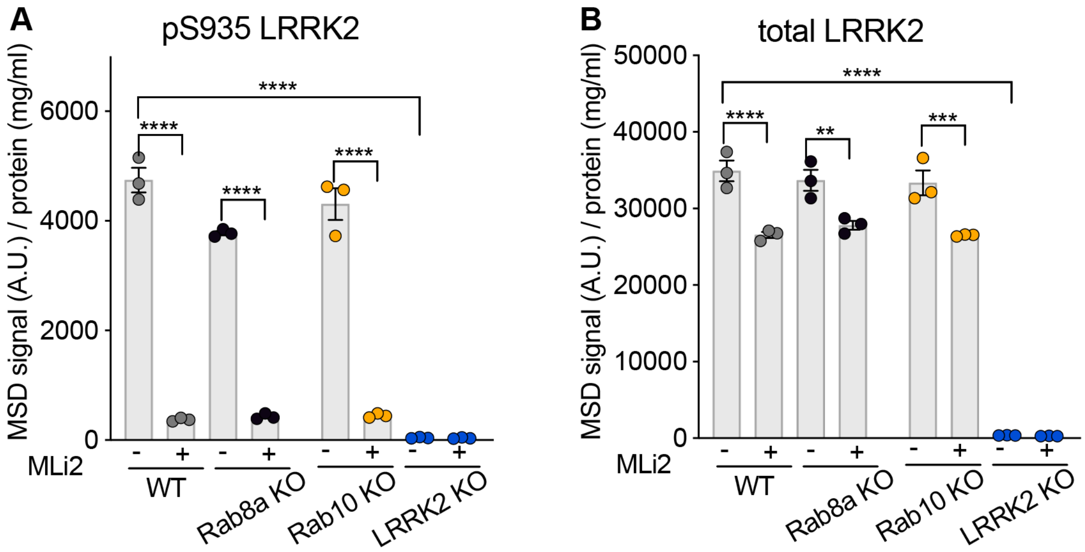

Supplementary Figure S3. pS935 LRRK2 and total LRRK2 levels measured by MSD assays in A549 cells across different genetic backgrounds.

A-B) pS935 LRRK2 and total LRRK2 levels were comparable in *RAB8A* or *RAB10* KO A549 cells and were absent in *LRRK2* KO cells. Data shown as mean ± SEM with p values: one-way ANOVA with Sidak's multiple comparison test. ** $p \leq 0.01$, *** $p \leq 0.001$, **** $p \leq 0.0001$.

Supplementary Figure S4

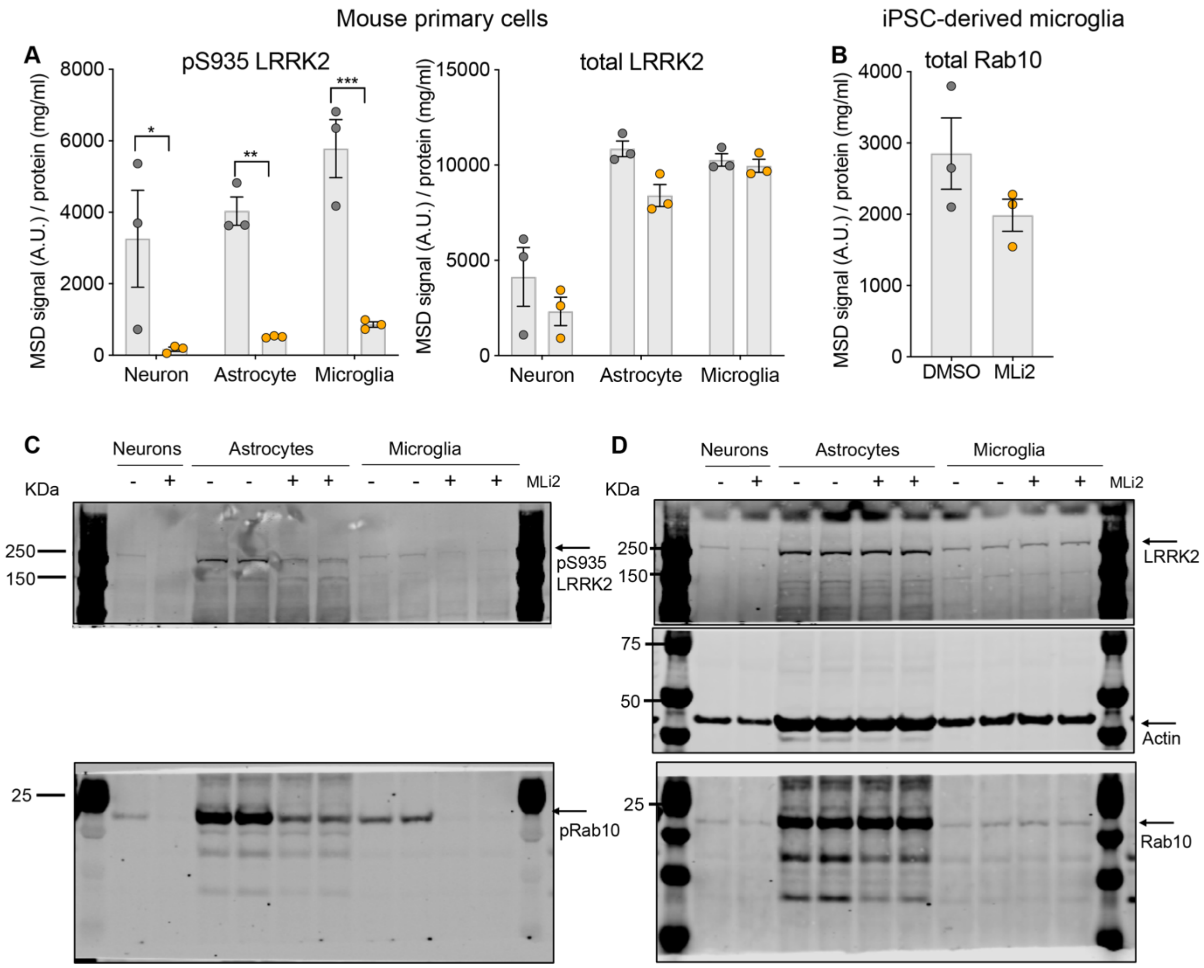

Supplementary Figure S4. LRRK2 is highly expressed in mouse glia cells

A) LRRK2 is highly expressed in mouse primary cultured cortical astrocytes and microglia, and inhibition of LRRK2 kinase by MLi-2 (500 nM, 2 hours) significantly reduced pS935 LRRK2. pS935 and LRRK2 levels were assessed by MSD assays; n=3. Data shown as mean ± SEM with p values: two-way ANOVA with Sidak's multiple comparison test. * $p \leq 0.05$, ** $p \leq 0.01$, *** $p \leq 0.001$. **B)** No significant difference in total Rab10 levels was observed following MLi-2 treatment (500 nM for 2 hours) in iPSC-derived microglia; n=3, Data are shown as mean ± SEM. **C-D)** The levels of LRRK2 and pRab10 in mouse primary cultured cortical neurons, astrocytes, and microglia, measured by western blot. pS935 LRRK2 and pRab10 were reduced with MLi2 (500 nM for 2 hours). The samples were run on NuPAGE 4-12% Bis-Tris gels, and the membranes were cut around 75 kDa and 25 KDa to enable analysis of multiple proteins. The top

membrane was blotted with rabbit anti-pS935 LRRK2 antibody (with secondary 800CW antibody) and mouse anti-LRRK2 antibody (with secondary 680RD antibody). The middle membrane was blotted with mouse anti-actin for a loading control (with secondary 680RD antibody). The bottom membrane was blotted with rabbit anti-pRab10 antibody (with secondary 800CW antibody), and mouse anti-Rab10 antibody (with secondary 680RD antibody). The left panel showed blot images in the 800 channel (**C**) and the right panel showed blot images in the 700 channel (**D**) taken using the LiCor odyssey imaging system.

Supplementary Figure S5

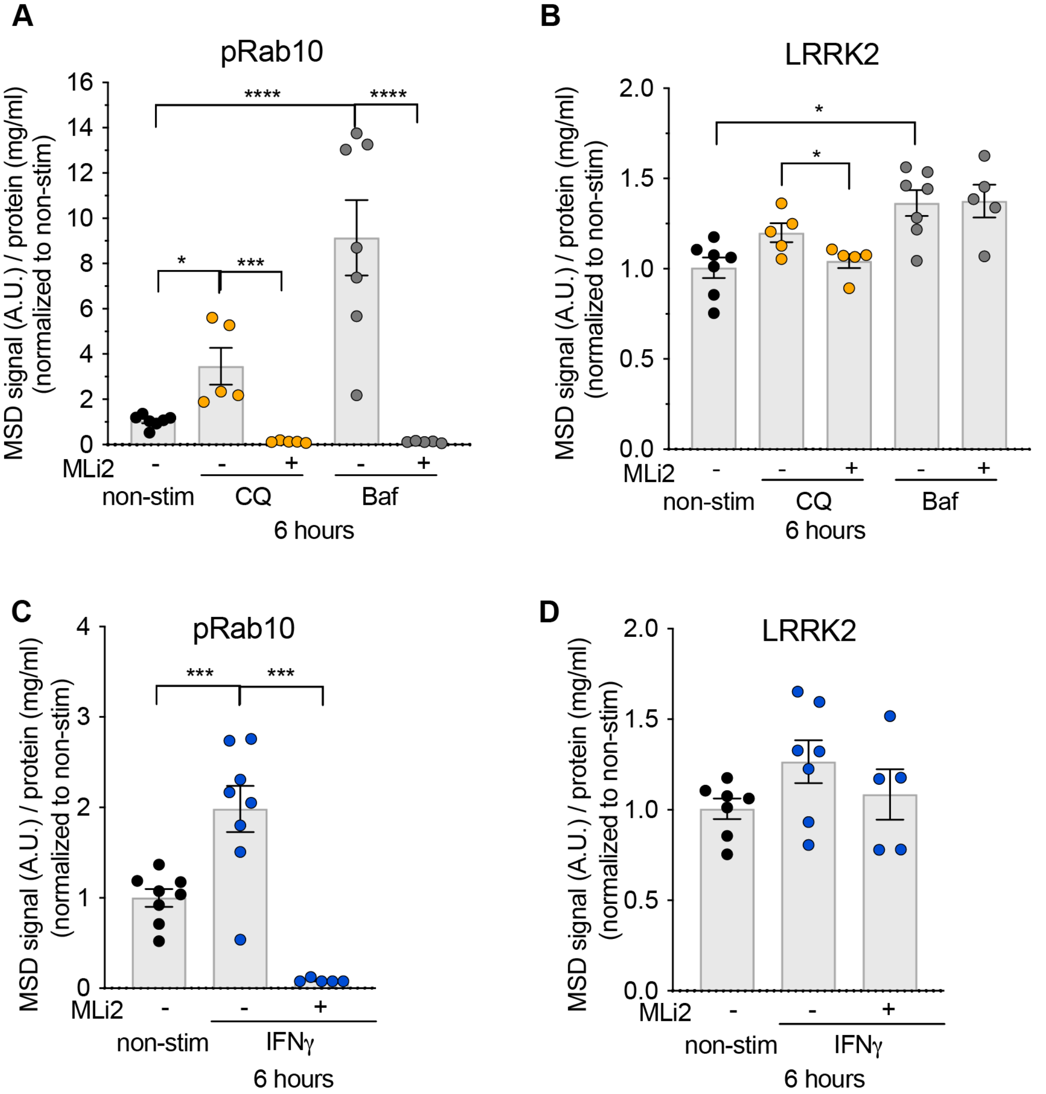

Supplementary Figure S5. Lysosomal stressors and inflammatory stimuli modulate LRRK2 levels and activity in iMicroglia.

A-B) In iMicroglia, acute treatment of chloroquine (50 µM for 6 hours) and bafilomycin A1 (100 nM for 6 hours) increased pRab10 levels, which were attenuated with LRRK2 kinase inhibitor (MLi-2, 500 nM) treatment. LRRK2 levels were largely not affected by the lysosomal stressors, with only a mild increase observed with Bafilomycin A1 treatment; N=5-8. **C-D)** Acute IFN- γ treatment (20 ng/mL, 6 hours) induced a significant increase in pRab10 levels in iMicroglia cells, with minimal effects on LRRK2 levels; n=5-8. Data were normalized to the median within each batch and then normalized to the control group; shown as mean ± SEM with p values analyzed

based on log transformed raw data using one-way ANOVA with Sidak's multiple comparison test. * $p \leq 0.05$, *** $p \leq 0.001$, **** $p \leq 0.0001$.

Supplementary Figure S6

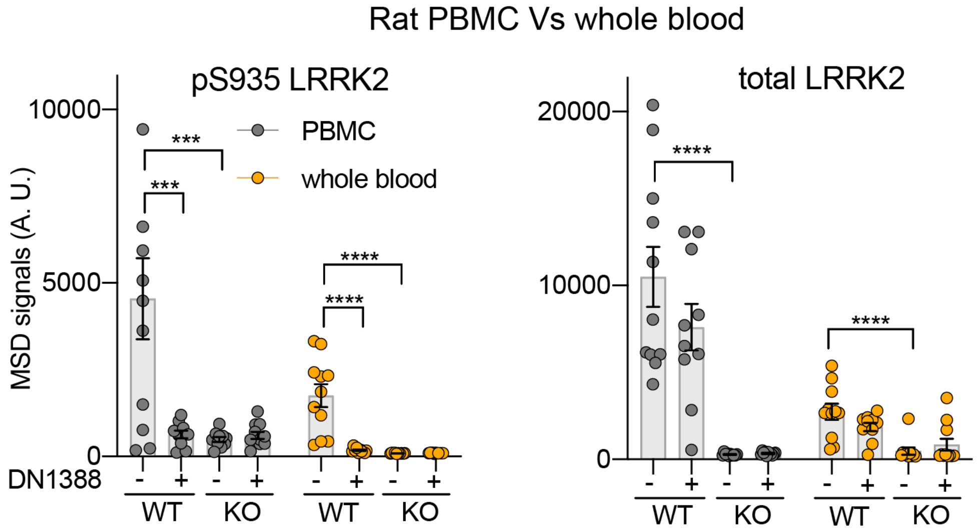

Supplementary Figure S6. Confirmation of specificity of pS935 LRRK2 and total LRRK2 MSD-based assays in rat PBMCs and whole blood.

pS935 LRRK2 and LRRK2 levels were measured in rat PBMCs and whole blood from WT and *LRRK2* KO rats with and without LRRK2 kinase inhibitor (DN1388, 100 mg/kg, QD, PO dosing for 10 days); n=11 animals/group. Data are shown as mean ± SEM with p values: two-way ANOVA with Tukey's multiple comparison test. *** $p \leq 0.001$, **** $p \leq 0.0001$.

Supplementary Figure S7

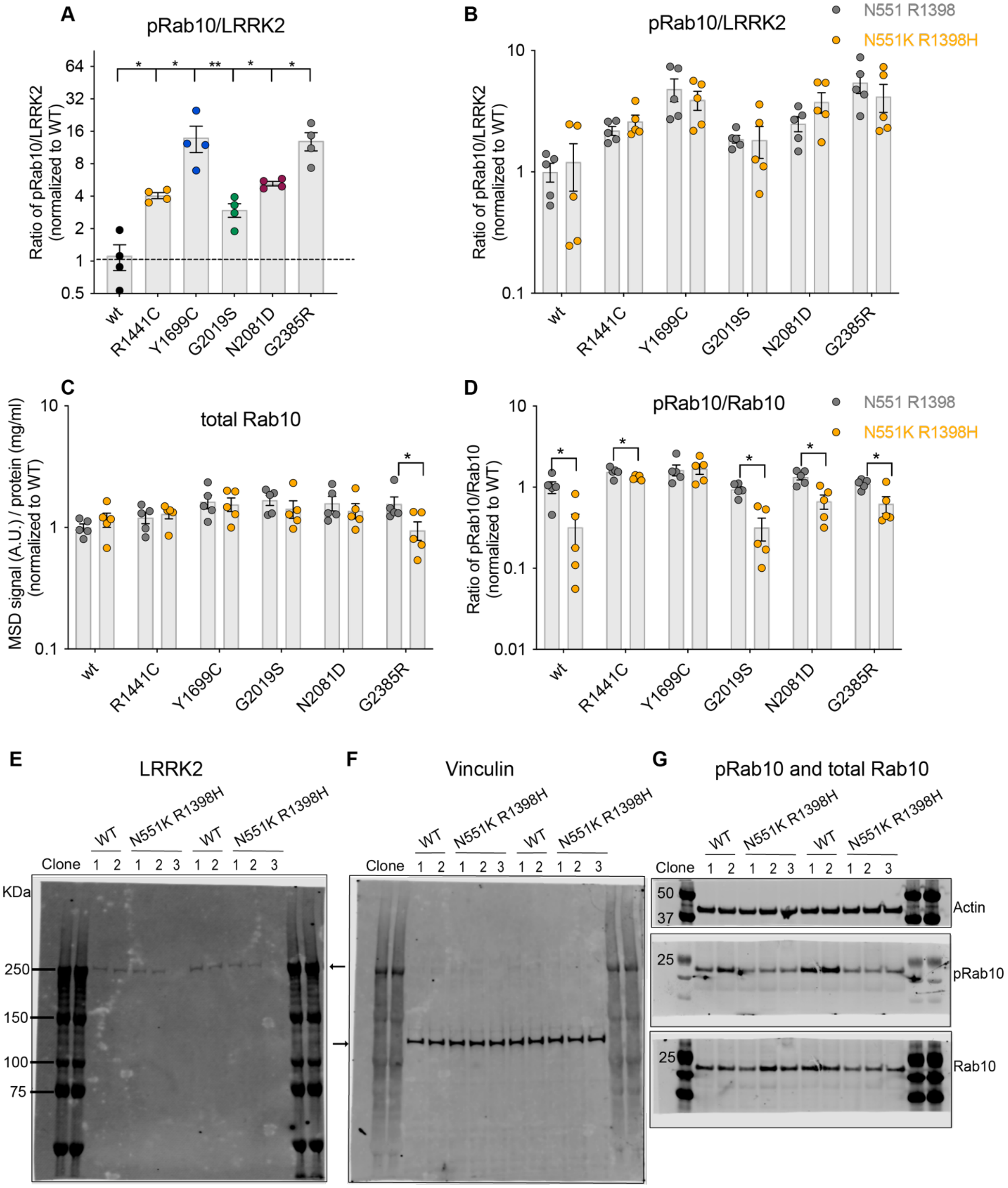

Supplementary Figure S7. Analysis of the effects of pathogenic and protective LRRK2 variants on LRRK2 and pRab10 levels in cellular models.

A) Expression of LRRK2 variants associated with increased risk for PD and Crohn's Disease lead to increased pRab10 levels (normalized to LRRK2) in HEK293T cells overexpressing LRRK2 and Rab10; n=4. **B-D)** In HEK293T cells overexpressing different LRRK2 variants and Rab10, total Rab10 levels and the ratio of pRab10/LRRK2 and the ratio of pRab10/Rab10 were analyzed. Data were normalized to the median within the batch, and then normalized to the wildtype group, shown as mean ± SEM. p values were analyzed based on log transformed data using repeated measures one-way ANOVA with Dunnett's multiple comparison test **(A)** or paired t-test **(C-D)**. * $p \leq 0.05$, ** $p \leq 0.01$. **E-G)** LRRK2 and pRab10 levels were reduced in LRRK2 N551K R1398H KI A549 cells as assessed by western blot analysis. **E and F**) To detect LRRK2 levels, samples were run on a NuPAGE 3-8% Tris-Acetate gel, and the protein transferred membrane was incubated with mouse anti-LRRK2 antibody and rabbit anti-vinculin. The same membrane image was shown at a high exposure for the LRRK2 channel (**E**) or at a low exposure for the vinculin channel as a loading control (**F**). **G**) To detect pRab10 and Rab10 levels, samples were run on a NuPAGE 4-12% Bis-Tris gel, and the membrane was cut around 50 kDa and 37 KDa. The top membrane was incubated with anti-actin antibody for a loading control, and the bottom membrane was incubated with rabbit anti-pRab10 antibody and mouse anti-total Rab10 antibody.

Supplementary Figure S8

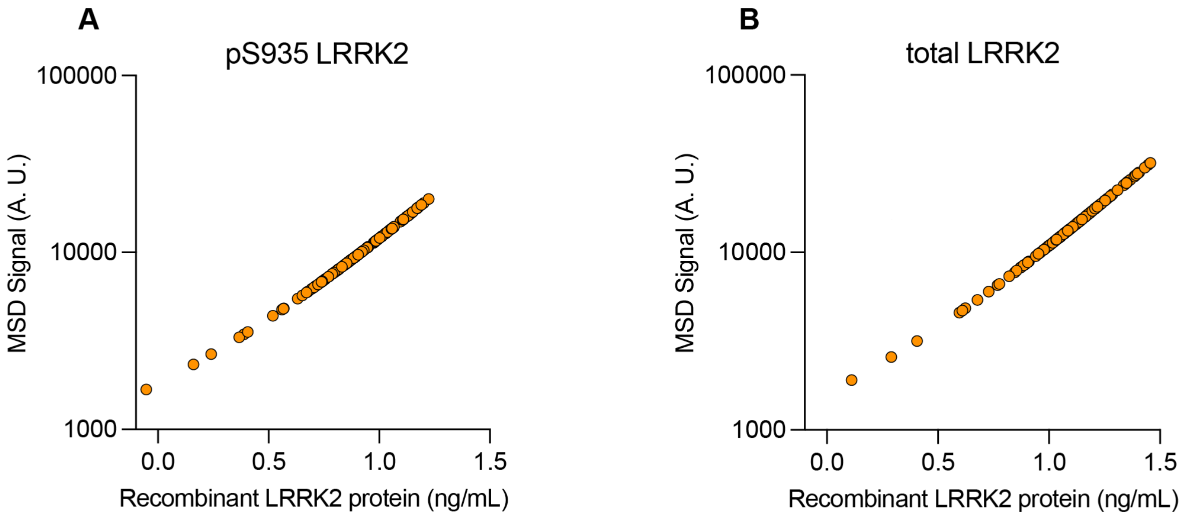

Supplementary Figure S8. The detection ranges of pS935 LRRK2 and LRRK2 in human PBMC lysates based on Figure 5A-B.

Supplementary methods

Western blot

Cell or tissue lysates were normalized for equal protein loading and were prepared by incubating with NuPage LDS Sample Buffer (ThermoFisher, NP0007) and NuPAGE™ Sample Reducing Agent (ThermoFisher, NP0004) for 10 min at 70 °C to denature samples. Lysates were loaded onto NuPAGE 4-12% Bis-Tris gels (Invitrogen) or NuPAGE 3-8% Tris-Acetate gels (Invitrogen). Proteins were transferred to nitrocellulose membranes for 7 min (Trans-Blot Turbo Transfer System, Bio-Rad). Some blots analyzed were cut prior to antibody incubations to enable the detection of several proteins, and the original cut and uncut blots are included in the Supplementary Figures. Membranes were blocked with Rockland blocking buffer, incubated with primary antibody overnight at 4 °C, and then with secondary antibodies (1:20,000, LI-COR) for 1 hour at room temperature. LI-COR Odyssey system was used for western blot detection and quantitation.

Genotype array data measurement, processing, and quality control

Samples used for the N551K R1398H genetic analysis were part of a dataset of 184 samples measured on the Illumina Infinium NeuroChip microarray platform (1). Whole genome amplification of DNA extracted from PBMCs, DNA fragmentation, two-step allele detection involving hybridization and single base extension were performed according to the manufacturer's instructions. Briefly, whole genome amplification occurred at 37°C for 20-24 hours, followed by enzymatic fragmentation at 37°C for 1 hour. The DNA was purified by alcohol precipitation, resuspended and hybridized on Illumina BeadChips at 48 °C for 16-24 hours. After single base extension and fluorescent labeling, the BeadChips were scanned using an Illumina iScan. Genotypes were called using the Neuro_Consortium_v1-1_20015375_A1.bpm annotation file (GRCh37 / hg19) via Illumina's GenomeStudio software. All steps from DNA extraction through genotype calling were performed by Q2 Solutions (Morrisville, NC, United States).

Prior to SNP-level and sample-level quality control carried out in Plink v1.9 (2) https://www.cog-genomics.org/plink/1.9/), genotype array data consisted of 487,374 variants measured on 184 samples. SNPs were removed if their Hardy-Weinberg Equilibrium p-value (estimated using control samples only) was < 1E-5, if their minor-allele frequency (MAF) was

less than 0.01, or if their call failure rate exceeded 5% of samples. Samples were removed if their call failure rate exceeded 5% of variants, and if they had outlying rates of heterozygosity (more or less than 3 SD from mean rate across all samples). To account for relatedness, we retained the sample with lower rate of genotype missingness among any pairs of samples in which the proportion of identity-by-descent metric (determined from LD-pruned data) exceeded 0.2. After these QC steps, genotype data was available on 302,591 variants and 174 samples.

Next we used the imputation preparation tool available from the McCarthy Group (https://www.well.ox.ac.uk/~wrayner/tools/) to perform strand flipping, reference/alternate allele assignment and other variant-level data checks prior to merging with 1000 Genomes (1000G) Phase 3 reference samples (3). We performed principal component analysis (PCA) on this merged dataset and used the first 10 principal components and 1000G super population labels to predict genetic ancestry for all samples in our dataset via the k-nearest neighbors algorithm. We then performed phasing using Eagle v2.4 (4) and imputed the data using reference panels from the predicted ancestry of the study samples. Imputation was performed using Minimac4 (5) with default parameters, and DosageConvertor (https://genome.sph.umich.edu/wiki/DosageConvertor) was used to convert Minimac4 output to Plink dosage format. Finally Plink v2 (2) https://www.cog-genomics.org/plink/2.0/) was used to convert the data to hard calls under the default parameters.

Statistical analysis of G2019S relationship to LRRK2, pS935 LRRK2, and pRab10 levels

For each variable, an analysis of covariance (ANCOVA) model was fit, with log2 transformed values as dependent variable and terms for disease status (PD vs Healthy controls), G2019S status (carrier vs non-carrier), and sex. A forward model selection step was performed to assess the usefulness of adjusting for age or including a Cohort by G2019 status interaction term in the model, with a significance level of 0.20 for inclusion of each of the additional terms in the model. Mean estimates of between group differences were back-transformed to the original scale and expressed as geometric means ratios (or relative geometric mean) and associated 95% confidence intervals.

Statistical analysis of N551K R1398H relationship to LRRK2 and pRab10 levels

Samples used for the N551K R1398H genetic analysis were part of a dataset of 184 samples measured on the Illumina Infinium NeuroChip microarray platform (1). Standard processing, quality control, and imputation procedures were implemented (see above for details). Data for the LRRK2 SNPs G2019S (rs34637584), N551K(rs7308720), and R1398H (rs7133914) were extracted from the genotype data and used in subsequent association analysis. While the G2019S and N551K variants were ascertained directly by the NeuroChip array, the R1398H variant was imputed. We verified that this variant was imputed to a high quality (Minimac4 (5), R-squared value > 0.99). Finally, we derived the N551K R1398H haplotype variable via the allele-level genotype data.

All statistical analysis was performed using R version 3.6.1. Of the 174 total samples with available imputed genotype data, 150 samples had complete data on the full set of covariates and MSD phenotypes used in association testing (see below) and were predicted to be of European ancestry. An additional 10 samples were removed due to outlying values (more than 3*SD from the mean across all samples for that phenotype) on 1 or more MSD phenotypes. Therefore, a total of 140 samples (including 14 N551K R1398H haplotype carriers) with joint genotype, MSD, and covariate data were available for association testing.

Raw protein levels for total LRRK2, pRab10 and pS935 LRRK2, along with the computed pRab10/LRRK2 ratio were natural log-transformed and fit in a linear model against sex, age, and the first two principal components ("Model 1", see below) of the measured genotype array data (to account for genetic ancestry). The residuals were then inverse-normal transformed using the blom() function from the 'rcompanion' package (https://cran.r-project.org/web/packages/rcompanion/index.html). These transformed residuals were then used in association testing against the N551K R1398H haplotype. Because only 2 samples were homozygous haplotype carriers, we collapsed heterozygous and homozygous carriers together and tested for association in carriers (n = 14) relative to non-carriers (n = 126). Finally, we carried out sensitivity analyses to evaluate to the impact of disease status ("Model 2"), LRRK2 G2019S status ("Model 3") or both ("Model 4") on the statistical inferences from Model 1.

References

1. C. Blauwendraat et al., NeuroChip, an updated version of the NeuroX genotyping platform to rapidly screen for variants associated with neurological diseases. Neurobiol Aging 57, 247.e249-247.e213 (2017).
2. C. C. Chang et al., Second-generation PLINK: rising to the challenge of larger and richer datasets. Gigascience 4, 7 (2015).
3. A. Auton et al., A global reference for human genetic variation. Nature 526, 68-74 (2015).
4. P.-R. Loh et al., Reference-based phasing using the Haplotype Reference Consortium panel. Nature Genetics 48, 1443-1448 (2016).
5. S. Das et al., Next-generation genotype imputation service and methods. Nat Genet 48, 1284-1287 (2016).
